# WS-RCNN: Learning to Score Proposals for Weakly Supervised Instance Segmentation

**DOI:** 10.3390/s21103475

**Published:** 2021-05-17

**Authors:** Jia-Rong Ou, Shu-Le Deng, Jin-Gang Yu

**Affiliations:** School of Automation Science and Engineering, South China University of Technology, Guangzhou 510641, China; au_jaring@mail.scut.edu.cn (J.-R.O.); audsl@mail.scut.edu.cn (S.-L.D.)

**Keywords:** weakly supervised learning, instance segmentation, proposal scoring network

## Abstract

Weakly supervised instance segmentation (WSIS) provides a promising way to address instance segmentation in the absence of sufficient labeled data for training. Previous attempts on WSIS usually follow a proposal-based paradigm, critical to which is the proposal scoring strategy. These works mostly rely on certain heuristic strategies for proposal scoring, which largely hampers the sustainable advances concerning WSIS. Towards this end, this paper introduces a novel framework for weakly supervised instance segmentation, called Weakly Supervised R-CNN (WS-RCNN). The basic idea is to deploy a deep network to learn to score proposals, under the special setting of weak supervision. To tackle the key issue of acquiring proposal-level pseudo labels for model training, we propose a so-called Attention-Guided Pseudo Labeling (AGPL) strategy, which leverages the local maximal (peaks) in image-level attention maps and the spatial relationship among peaks and proposals to infer pseudo labels. We also suggest a novel training loss, called Entropic OpenSet Loss, to handle background proposals more effectively so as to further improve the robustness. Comprehensive experiments on two standard benchmarking datasets demonstrate that the proposed WS-RCNN can outperform the state-of-the-art by a large margin, with an improvement of 11.6% on PASCAL VOC 2012 and 10.7% on MS COCO 2014 in terms of mAP${}_{50}$, which indicates that learning-based proposal scoring and the proposed WS-RCNN framework might be a promising way towards WSIS.

## 1. Introduction

Instance segmentation [1,2,3,4] refers to the task of jointly localizing, categorizing and segmenting the spatial extents of individual visual objects from a given image. Like many other computer vision tasks, remarkable progress has recently been made on instance segmentation driven by the prosperity of convolutional neural networks (CNN) [2,4,5,6,7]. Nevertheless, CNN-based solutions to vision tasks commonly suffer from the data-hungry nature, i.e., the necessity of a large amount of annotated data for training. This may be particularly infeasible for instance segmentation since pixel-wise annotations at the instance level are extremely labor-intensive. Weakly supervised instance segmentation (WSIS)  [8,9] is one possible way to alleviate the dependency on such strong annotations, which aims to achieve instance segmentation by the use of weaker and thus less labor-intensive annotations [10,11,12], ideally image-level labels only [8] as we are concerned with in the present work.

There recently emerge a couple of attempts on WSIS with image-level labels in the literature [8,9,13,14,15], which can be typically outlined as three major steps: (1) proposal generation, i.e., a number of class-agonistic segment proposals are generated from the given image; (2) proposal scoring, i.e., classification scores are assigned to the proposals; (3) postprocessing, i.e., final results are retrieved from the scored proposals by using non-maximal suppression or certain postprocessing procedures (e.g., applying Mask R-CNN for refinement [14]). It has been well-established that CNN classifiers trained globally at the image level have remarkable ability of spatial localization [16,17], and  the so-called attention maps of a certain form (e.g., Class Activation Maps [18], Occlusion Maps [19], Saliency Maps [20], Excitation Backprop [17]) are utilized to represent such localization cues, where the intensity stands for the possibility of spatial occurrence of visual objects. Taking advantage of this fact, previous works on WSIS mainly put their efforts on the step of proposal scoring, i.e., how to infer reasonable classification scores for the proposals from global attention maps. As a pioneering work on WSIS, Zhou et al. [8] proposed Peak Response Map (PRM) to boost the instance localization ability of CNNs, which then enables better proposal scoring. Zhu et al. [13] presented the Instance Activation Map which aims to enhance the spatial extents of instances in PRM and consequently improves proposal scoring. Ahn et al. [14] deployed the Inter-pixel Relation Network to learn a high-quality proposal generator, as well as pairwise semantic affinities which guide the proposal scoring.

**Figure 1:**
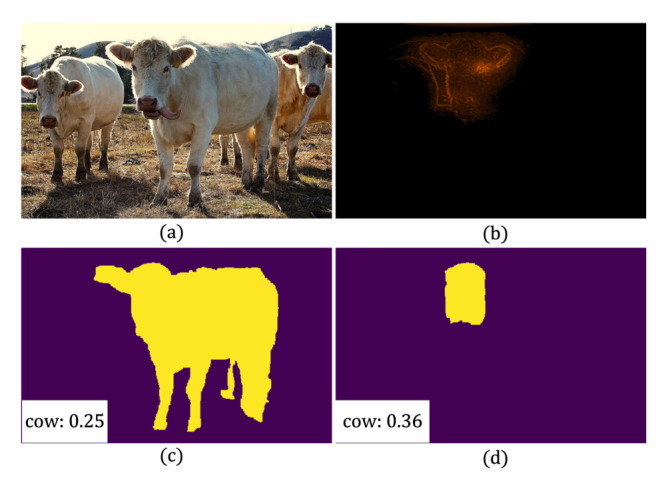
Illustration of the motivation of our work. Existing proposal-based approaches to WSIS commonly follow a heuristic way to exploit attention maps for proposal scoring. In order to assign high scores to the proposals of true objects, the attention maps are expected to be aware of the spatial extents of objects, which is also the main focus of previous efforts on WSIS. But unfortunately, this is by nature a very difficult perceptual grouping task, since attention maps can only have very coarse resolution sparsely highlighting some discriminative sites (unaware of object extents). For intuition, we show some exemplary results obtained by the poineering approach of PRM [8], where (**a**) is the original image, (**b**) is the PRM of the *cow* class, and (**c**,**d**) are two proposals and the obtained classification scores. As can be seen, the more favorable proposal in (**c**) is undesirably assigned with a lower score.

While these wisdoms of making improvements on proposal scoring (or other particular components) can indeed benefit to an extent, previous works on WSIS share one major limitation that they commonly follow a heuristic way to exploit attention maps for proposal scoring, lacking of a unified framework. Concretely, they utilize certain attention maps and hand-crafted scoring rules to assign classification scores to proposals [8,13]. In this way, the attention maps are expected to be aware of the spatial extents of visual objects, such that they can match the shapes of true objects and consequently assign them with high scores. On the other hand, attention maps can only sparsely highlight some sites discriminative for classification, usually at very coarse spatial solutions and unaware of object extents. Such discrepancy explains why previous works all devote their efforts to enhancing the perception of object extents of attention maps. Nevertheless, this is by nature a rather difficult perceptual grouping task (see Figure 1), which may limit the substantial advances on this topic. These observations motivate us to consider if these exist a simpler but more effective way towards WSIS.

In this paper, we introduce a general framework for weakly supervised instance segmentation, called Weakly Supervised R-CNN (WS-RCNN). The underlying idea is very simple and natural: Instead of relying on heuristic strategies for proposal scoring, we deploy a deep network to learn to score proposals. For this purpose, there exists an inherent challenge, i.e., how to acquire proposal-level pseudo labels to enable the learning since only image-level labels are available in our problem setting (the pseudo labeling issue)? This is actually a key issue for any weakly supervised learning problem including ours. In addition, an appropriate training loss function is also well worthy of exploration in our learning task. We propose specific solutions to these key issues and further design a unified framework for WSIS. Since the resulting framework can be conceptually interpreted as a Fast R-CNN model [21], which is a representative model of general object detection among the R-CNN family [2,21,22,23], under the particluar setting of weak supervision, we name our framework as Weakly Supervised R-CNN (WS-RCNN).

As another major contribution, we propose an effective solution to this pseudo labeling issue, termed as Attention-Guided Pseudo Labeling (AGPL). AGPL also utilizes attention maps, however it relies only on local maxima (peaks) in attention maps and the spatial relationship among peaks and proposal regions. Specifically, it admits object instances of a target class by inspecting the positional inclusion relationship between proposals and peaks in the attention maps associated with that particular class (see Section 3.3 for details). The key insight lies in that AGPL leverages very weak information, instead of necessitating to take into account object extents like existing approaches [8,9,13,14], which makes the pseudo labeling procedure simple but robust. We stress that such careful design of AGPL is crucial to the success of the whole WS-RCNN framework (see Section 4.3 for experimental validation).

As our third contribution, we suggest a novel loss function for model training to further boost the performance, called Entropic Open-Set (EOS) Loss. As our method is proposal-based, the proposal generator will inevitably yield a portion of background proposals (those belonging to none of the concerned classes) even with the state-of-the-art method. How to make our model conscious of background proposals to avoid misclassification is important to its robustness. This is by nature an open-set recognition problem [24,25], which has been well studied in the context of robust pattern recognition. We propose to adapt the state-of-the-art method for CNN-based open-set recognition [25] to our background handling. To our knowledge, this is the work which considers the open-set issue in object detection/instance segmentation.

We will carry out comprehensive experiments on standard benchmarking datasets to demonstrate that WS-RCNN can push forward the state-of-the-art on WSIS by a remarkable margin, with an improvement of 11.6% on PASCAL VOC 2012 and 10.7% on MS COCO in terms of ${\mathrm{mAP}}_{50}$ (see Section 4.2 for details). Such overwhelming superiority in performance suggests that WS-RCNN might be a more appropriate framework for WSIS worthy of further exploration than existing ones which focus on deducing object extents from attention maps for proposal scoring. To sum up, the main contributions of our work are as below:

- We propose a novel framework called Weakly Supervised R-CNN (WS-RCNN) for weakly supervised instance segmentation. The key insight is to deploy a deep network to learn to score proposals, instead of relying on heuristic proposal scoring strategies, which may provide a new perspective for future exploration on WSIS.
- We propose a simple but effective strategy for inferring pseudo labels from attention maps, called Attention-Guided Pseudo Labeling (AGPL).
- We introduce an Entropic Open-Set (EOS) Loss for handling background proposals in model training to further boost the performance.

The remainder of this paper is organized as follows: Section 2 briefly reviews related literature. Section 3 details the proposed WS-RCNN framework, including the AGPL scheme for pseudo labeling and the EOS loss. Section 4 focuses experiments, including comparison, ablation study and related analysis. In Section 5, we make a conclusion and give some remarks on future work.

## 2. Related Work

In this section, we present a brief review of the literature that are closely related to ours, including those on weakly supervised instance segmentation, weakly supervised semantic segmentation, weakly supervised object detection and open-set recognition and background handling.

### 2.1. Weakly Supervised Instance Segmentation

Instance segmentation aims to produce accurate masks that can distinguish between instances of specific object classes. It generally requires pixel-wise object masks to train an instance segmentation model, which are extremely expensive. Weakly supervised instance segmentation (WSIS) devotes to conquering this challenge by using certain forms of weaker annotations, most ideally image-level labels only as concerned in this paper. Along the line of WSIS using image-level annotations, PRM [8] is a pioneering work which treats peaks in class response maps obtained by CNN classifiers as indicators of the existence of object instances, and introduces the so-called Peak Response Map (PRM) to score object proposals so as to identify true object instances. In [26], the results obtained by PRM are used as pseudo annotations to train a Mask R-CNN [2] for further refinement. Zhu et al. [13] proposed the Instance Activation Map to enhance PRM [8] by taking better care of spatial extents of objects. Ahn et al. [14] presented the Inter-pixel Relation Network to learn a good proposal generator, and also inter-pixel connections which can guide proposal scoring. In [9], the authors collaboratively combined weakly supervised object detection with WSIS under a unified framework of course learning. As previously stated, WSIS generally remains at the early stage of exploration, and the few existing approaches mainly focus on figuring out the shapes of object instances from attention maps for better proposal scoring, which faces a very difficult perceptual grouping task.

### 2.2. Weakly Supervised Semantic Segmentation

The mainstream paradigm for weakly supervised semantic segmentation (WSSS) can be summarized as to derive pseudo semantic masks to enable the training of a model of fully supervised semantic segmentation (most typically FCN [27]), and existing approaches can be distinguished by how they acquire the pseudo masks. In [28], discriminative regions are selected from CAMs [18] based on three principles, called “seed, expand and constrain”, as pseudo labels to supervise a segmentation network. Roy et al. [29] utilized a novel deep architecture which fuses bottom-up, top-down, and smoothness cues to acquire pseudo masks. Other representative strategies for enhancing the quality of pseudo masks include the LSE pooling [30], the EM algorithm [31], seeded region growing [32], semantic affinity [33], dilated convolution [34], the anti-erase strategy [35,36], similar region mining [37], the self-erasing strategy [38], visual saliency [39,40], etc. Although WSSS is another task different from WSIS, our work was somewhat inspired by the paradigm of inferring pseudo labels to train fully supervised models.

### 2.3. Weakly Supervised Object Detection

The task of weakly supervised object detection (WSOD) is similiar to WSIS except for the necessity of yielding instance segmentation masks. Earlier works on WSOD mostly follow the pipeline of multiple instance learning [41,42,43]. Recently CNN-based approaches have attracted more and more research interest. As a milestone work along this direction, WSDDN [44] adopts a two-branch network where softmax operations are performed over proposals and classes respectively in the two branches, and the obtained classification scores are synthesized into an image-level score so as to establish the training loss. Diba et al. [45] introduced a novel cascaded network for WSOD, which performs ROI pooling at multiple levels to boost the performance. Ref. [46] proposes a weakly supervised region proposal network which is trained using only image-level annotations and the proposed region proposal network can be plugged into a WSOD network easily. In [47], Tang et al. introduced the Proposal Clustering Learning which generates proposal clusters to learn refined instance classifiers by an iterative process. Other successful approaches to WSOD include the Min-Entropy Latent Model [48], the  Category-Aware Spatial Constraint [49], etc. While the tasks are essentially different, these works on WSOD are similar to ours in being proposal-based.

### 2.4. Open-Set Recognition and Background Handling

Traditional recognition systems obey the closed-set assumption, i.e., the training and testing data are drawn from the the same set of semantic classes. However, a more realistic scenario is that data from unseen classes can emerge unexpectedly during testing, which may drastically decrease the robustness of the system. This is well-known as the open-set issue in pattern recognition, which has attracted a lot of research interests [24,25,50]. In two-stage object detectors (like FRCNN [21] and our WS-RCNN), the proposals obtained by the proposal generator will inevitably include a portion of proposals from image background (unseen classes to the model), which is by nature an open-set recognition problem [24]. But surprisingly, no previous authors have addressed this problem from the perspective of open-set recognition to our knowledge. Instead, they usually perform background handling in certain ad-hoc strategies, e.g., adding a dummy background class to the model.

## 3. Approach

In this section, we start with the problem statement of WSIS, followed by describing the proposed WS-RCNN framework. Then we detail the key components, including the Attention-Guided Pseudo Labeling and the Entropic Open-Set Loss. Finally, we present some remarks.

### 3.1. Problem Statement

Given a set of classes of interest C={1,…,C} and a training set $I={\left\{\left(I_{k},y_{k}\right)\right\}}_{k=1}^{K}$, where $I_{k}$ is an image and $y_{k}\in {\left\{0,1\right\}}^{C\times 1}$ is the corresponding multi-class label vector, the task of weakly supervised instance segmentation (WSIS) in our work can be roughly stated as to segment, for an input testing image, all the object instances belonging to the classes C. Such a problem setting differs intrinsically from general instance segmentation [2,4] in that no pixel-wise instance annotations but only image-level labels are available for model establishment, which makes the task very challenging.

Like general instance segmentation, WSIS can also follow a proposed-based paradigm [8,13,33], which can be epitomized as a three-step pipeline as aforementioned. These approaches can then be viewed as to retrieve true object instances from a pool of proposals according to the assigned scores, central to which is proposal scoring, i.e., how to appropriately assign classification scores to proposals. One commonly-used strategy for proposal scoring is to make use of the well-established localization ability of CNNs [8,16,18]. Specifically, the training set I with image-level labels are firstly taken to train an image-level CNN classifier, from which a collection of class-specific attention maps are derived to assign classification scores to the proposals. For this purpose, it is desired that these attention maps can preserve object shapes, which is however a difficult perceptual grouping task. In addition, the hand-crafted scoring rules adopted by existing methods are also limited as well. These facts motivates us to propose the WS-RCNN framework.

### 3.2. The Proposed WS-RCNN Framework

The basic idea of WS-RCNN is to deploy a deep network to learn to score proposals under the special setting of weak supervision, instead of relying on heuristic proposal scoring strategies. To achieve this goal, one major obstacle is the absence of proposal-level labels necessitated for training. To conquer this challenge, we develop an effective strategy, called Attention-Guided Pseudo Labeling (AGPL), to take advantage of the attention maps associated with the image-level CNN classifier to infer proposal-level pseudo labels. Furthermore, we introduce an Entropic Open-Set Loss (EOSL) to handle the background issue in training to further improve the robustness of our framework. In the following, we will first present an overview of WS-RCNN, followed by detailing the AGPL stategy and the EOSL loss.

**Figure 2:**
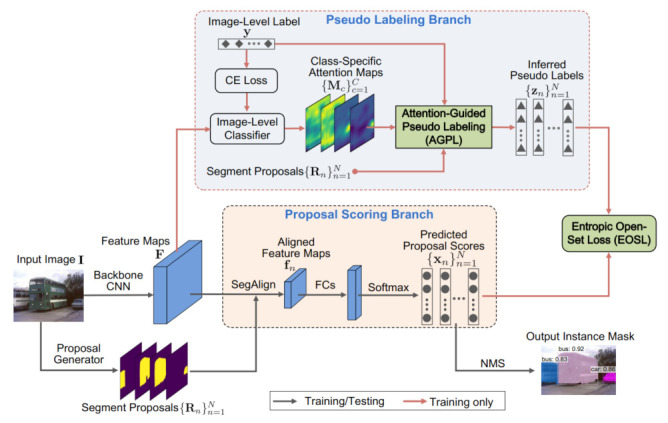
Overview of the proposed Weakly Supervised R-CNN (WS-RCNN) framework for weakly supervised instance segmentation. WS-RCNN adapts Fast R-CNN (FRCNN) [21], a representative model for general object detection, to the particular setting of weak supervision, which can be roughly interpretted as to derive pseudo labels from the image-level CNN classifier to initiate the FRCNN. The network mainly consists of two streams, i.e., the proposal scoring stream and the image-level classification stream. The former learns to score proposals, trained by the Entropic Open-Set Loss, and the latter performs pseudo labeling using the Attention-Guided Pseudo Labeling.

Network Architecture: The overall network architecture of WS-RCNN is shown in Figure 2. Following the notations above, the input image I sized by $H_{I}\times W_{I}$ is first fed into a proposal generator (using the off-the-shelf method [51,52] in our implementation) to obtain the segment proposals ${\left\{R_{n}\right\}}_{n=1}^{N}$, where each $R_{n}$ is an $H_{I}\times W_{I}$ binary mask representing a segment proposal with arbitrary shapes (rather than regular bounding-boxes). The image then goes through a backbone CNN for feature extraction, yielding the feature maps $F\in R^{H\times W\times M}$, where H×W is the size and *M* the number of the feature maps. Afterwards, the network bifurcates into two branches, i.e., the proposal scoring branch and the pseudo labeling branch. Notice that these two branches share the same backbone CNN.

In the proposal scoring branch, the features corresponding to each individual proposal are extracted. A standard operation for this task is RoIAlign [2], widely used in two-stage object detectors, which however cannot be directly applied to our case since it is designed for bounding-box proposals. Therefore, we modify RoIAlign to adapt to segment proposals, resulting in the SegAlign operation (see details below). For each proposal $R_{n}$, the corresponding features can be extracted from F and aligned to a canonical grid via SegAlign, denoted by $f_{n}\in R^{h\times w\times M}$ (we use h = w = 7; M = 512 in this paper), which is followed by three fully-connected layers (FCs, with the node numbers being 4096, 4096 and C respectively) and a softmax layer to get the proposal-level classification score $x_{n}\in R^{C\times 1}$.

The pseudo labeling branch is executed for training only, where the feature maps F are followed by an image-level classifier. Then, a set of class-specific attention maps, denoted by ${\left\{M_{c}\right\}}_{c=1}^{C}$, are extracted from this classifier, where each $M_{c}$ reflects the spatial probability of occurrence of object instances belonging to the class c. Among possible choices of attention maps, we adopt the Class Peak Responses [8] in our implementation due to its excellent localization ability. These attention maps (as well as the image-level label y) are then utilized to infer the proposal-level pseudo class labels ${\left\{z_{n}\right\}}_{n=1}^{N}$, where $z_{n}\in R^{C\times 1}$ is a one-hot vector $z_{n}\in 0^{C\times 1}$ standing for the background class), by the use of AGPL.

Training Strategy: We adopt a two-phase training strategy to train the WS-RCNN model. In the first phase, we train the image-level classifier in the pseudo labeling branch, which is initialized by the model pre-trained on ImageNet. Proposal-level pseudo labels are then inferred from the trained imagelevel classifier using AGPL. In the second phase, we train the proposal scoring branch, where the backbone CNN is reinitialized with the model pre-trained on ImageNet. We will validate the effectiveness of this two-phase training strategy by comparative experiments in Section 4.3. Notice that since there usually exist significantly more background proposals than target-class ones after pseudo labeling, we always make their numbers identical by uniformly sampling background proposals.

Training Loss: For the training of the image-level classifier (the first-phase training), we use the given image-level labels ${\left\{y_{k}\right\}}_{k=1}^{K}$ and the conventional cross-entropy loss function for multi-label classification to establish the training loss.

For the training of the proposal scoring branch (the secondphase training), suppose for the image $I_{k}$ labeled with $y_{k}$ in the training set I, the proposals obtained are ${\left\{R_{n}^{k}\right\}}_{n=1}^{N}$. For each $R_{n}^{k}$, let us denote by $x_{n}^{k}$ the classification score predicted by the proposal scoring branch, and by $z_{n}^{k}$ n the pseudo class label inferred by the pseudo labeling branch using AGPL. Given all these, the training loss for the proposal scoring stream can be established by

(1) $$L_{p}=\frac{1}{K}\sum_{k=1}^{K}\left[\frac{1}{N}\sum_{n=1}^{N}\ell_{\mathrm{EOSL}}\left(x_{n}^{k},z_{n}^{k}\right)\right],$$

where $\ell_{\mathrm{EOSL}}$ is the proposed Entropic Open-Set Loss (see details in Section 3.4).

**Figure 3:**
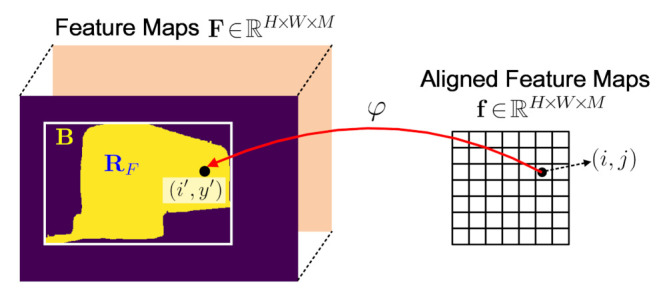
Illustration of the SegAlign operation. SegAlign adapts the widely-used RoIAlign [2] to segment masks.

SegAlign: As shown in Figure 3, following the notations above, suppose for a segment mask R in the $W_{I}\times H_{I}$ image I, the corresponding receptive field mapped to the feature maps $F\;\in \;R^{H\times W\times M}$ is $R_{F}$. SegAlign extracts from F the features corresponding to $R_{F}$ and maps them to canonical feature maps $f\;\in \;R^{h\times w\times M}$, which is basically a modified RoIAlign to adapt to segment masks. Concretely, suppose $R_{F}$ is bounded by the rectangle B, φ is the bilinear transform from the spatial coordinates (i,j)∈f to $(i^{\prime },j^{\prime })\in B$, i.e., $\left(i^{\prime },j^{\prime }\right)=\varphi \left(i,j\right)$, and  *g* is the bilinear interpolation function over F. The SegAlign operation can then be defined by

(2) $$f\left(i,j\right)=\left\{\begin{array}{c} g\left(F,\varphi \left(i,j\right)\right),\;\;\mathrm{if}\;\;\varphi \left(i,j\right)\in R_{F},\\ 0,\;\;\mathrm{otherwise}. \end{array}\right.$$

Note we drop the channel dimension of feature maps above without loss of clarity.

### 3.3. Attention-Guided Pseudo Labeling

**Figure 4:**
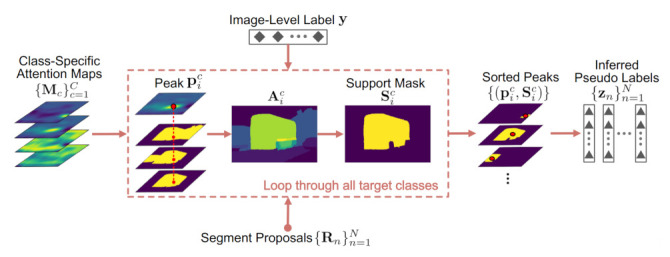
Illustration of Attention-Guided Pseudo Labeling (AGPL). AGPL leverages the localization ability of CNNs and the spatial relationship among proposals to achieve pseudo labeling.

AGPL leverages the localization ability of CNNs and the spatial relationship among proposals to achieve pseudo labeling. As shown in Figure 4, for the image I∈I, given the class label vector $y={\left[y_{1},\ldots ,y_{C}\right]}^{T}$, the segment proposals ${\left\{R_{n}\right\}}_{n=1}^{N}$ and the class-specific attention maps ${\left\{M_{c}\right\}}_{c=1}^{C}$, AGPL can be outlined as follows:

(1) For each target class *c* (with $y_{c}=1$), all the local maxima (peaks) are identified from $M_{c}$, denoted as ${\left\{p_{i}^{c}\right\}}_{i=1}^{m_{c}}$, where $p_{i}^{c}\in R^{2}$ stands for pixel coordinates and $m_{c}$ the number of peaks. For each $p_{i}^{c}$, we pick up all the proposals spatially including this point, which are further averaged and thresholded to get a support mask $S_{i}^{c}$ as follows

(3) $$A_{i}^{c}=\frac{1}{m_{i}^{c}}\sum_{\left\{n|p_{i}^{c}\in R_{n}\right\}}\;\;R_{n},$$

(4) $${\left(S_{i}^{c}\right)}_{pq}=\left\{\begin{array}{cc} {\left(A_{i}^{c}\right)}_{pq},\;\; & \mathrm{if}\;\;{\left(A_{i}^{c}\right)}_{pq}>\beta ,\\ 0,\;\; & \mathrm{otherwise}. \end{array}\right.$$

where $m_{i}^{c}$ is the number of picked proposals corresponding to $p_{i}^{c}$, *p* and *q* are pixel indices, and the threshold β∈[0,1] is a parameter (we adopt β=0.7 in our implementation. See Section 4.4 for parameter study). The resulting peaks ${\left\{p_{i}^{c}\right\}}_{i=1}^{m_{c}}$ and associated support masks ${\left\{S_{i}^{c}\right\}}_{i=1}^{m_{c}}$ are then utilized to admit proposals belonging to the class *c*.

(2) Sort all the peaks $\left\{p_{i}^{c}|c=1,\ldots ,C;i=1,\ldots ,m_{c}\right\}$ in the descending order of their values in the attention maps, i.e., $\left\{M_{c}\left(p_{i}^{c}\right)\right\}$. Then, for each ordered peak $p_{i}^{c}$ and the associated $S_{i}^{c}$, those proposals which overlap sufficiently with $S_{i}^{c}$ are labeled as the class *c*, i.e., $z_{n}=c$ if

(5) $$\mathrm{IoU}(S_{i}^{c},R_{n})>0.5,$$

where IoU stands for the Intersection-over-Union operation. Notice that one proposal is allowed to be exclusively assigned to one class only during the ordered labeling. For clarity, we summarize the AGPL algorithm above in Algorithm 1.

### 3.4. Entropic Open-Set Loss

Since our WS-RCNN is proposal-based, the proposals after pseudo labeling will unavoidably contain some background proposals, i.e., those labeled as none of the target classes. It is necessary to handle these background proposals in model training, otherwise a model trained only with samples from target classes will be distracted by the unseen background proposals in testing, degrading its robustness. A natural solution is to add a dummy class into the model to accommodate background proposals. However, since the background class is a class of “stuff”, its variance is so large that it is hard to be modeled by any single class.
**Algorithm 1** Attention-Guided Pseudo Labeling (AGPL) **Input: **The label vector $y=\left[y_{1},\ldots ,y_{C}\right]\in {\left\{0,1\right\}}^{C\times 1}$, the segment proposals ${\left\{R_{n}\right\}}_{n=1}^{N}$ and the class-specific attention maps ${\left\{M_{c}\right\}}_{c=1}^{C}$ (associated with the image I); the parameter β. **Output:** The one-hot pseudo class labels ${\left\{z_{n}\right\}}_{n=1}^{N}$ for the proposals.1:Initialize $z_{n}=0$, Q←∅.2:**for all***c* with $y_{c}=1$ (target class) **do**3:  Find the local maxima ${\left\{p_{i}^{c}\right\}}_{i=1}^{m_{c}}$ in $M_{c}$;4:  **for** $i=1,\ldots ,m_{c}$ **do**5:    Calculate the support mask $S_{i}^{c}$ using Equations (3) and (4);6:    $Q\leftarrow (p_{i}^{c},S_{i}^{c})$;7:  **end for**8:**end for**9:Sort Q in the descending order of the values $\left\{M_{c}\left(p_{i}^{c}\right)\right\}$, denoted by $\tilde{Q}$ the sorted set.10:L←{1,…,N};11:**for all**$\left(p_{i}^{c},S_{i}^{c}\right)\in \tilde{Q}$**do**12:  Find the proposals indexed by I⊆L satisfying Equation (5);13:  Set ${\left(z_{n}\right)}_{c}=1,\;\;\forall n\in I$;14:  L←L\I;15:**end for**

To address this issue, we observe that the task of background handling here is by nature an open-set recognition (OSR) problem, which has been well studied in robust pattern recognition. Hence, we propose to introduce the Entropic Open-Set Loss (OSEL), which is a representative method for OSR [25], to address our background handling problem. The basic idea of OSEL is to treat the samples from target and background classes separately in establishing the training loss. For target classes, the standard cross-entropy loss is used, while for the background class, an entropic loss is used to encourage predicting uniformly-distributed classification scores. Since the *C* scores sum up to 1 (output by the softmax layer), encouraging uniform distribution on background class will make these scores small and therefore suppressed during the Non-maximal Suppression (NMS) procedure. Formally, suppose the predicted score vector of a proposal is $x=\left[x_{1},\ldots ,x_{C}\right]\in R^{C\times 1}$ and the corresponding one-hot pseudo label vector is $z=\left[z_{1},\ldots ,z_{C}\right]\in {\left\{0,1\right\}}^{C\times 1}$, the EOSL is defined by

(6) $$\ell_{\mathrm{EOSL}}\left(x,z\right)=\left\{\begin{array}{c} -z_{c}\log x_{c},\;\;\mathrm{if}\;z_{c}\neq 0,\\ -\sum_{i=1}^{C}\log x_{i},\;\;\mathrm{if}\;z=0. \end{array}\right.$$

To our knowledge, this is the first work which addresses the background handling problem in object detection/instance segmentation from the perspective of open-set recognition.

### 3.5. Remarks

Despite the proposed WS-RCNN is very natural and simple, we will later experimentally demonstrate that it has overwhelming superiority in performance over the state-of-the-art methods, which is very likely due to two key insights of our approach. First, we adopt a learning-based approach to proposal scoring, which is advantageous in being able to learn to directly map feature representations of proposals to classification scores, rather than depending on hand-crafted scoring rules. Second, AGPL only utilizes very loose information to achieve pseudo labeling, i.e., some sparse points (peaks in attention maps) and their spatial relationship with proposal regions, which makes our approach much easier and thereby more robust to complex scenes in comparison with existing works that involve the consideration of spatial extents of objects. The proposed WSRCNN framework can be simply interpreted as “learning to score proposal under weak supervision”, which may provide a promising new perspective for addressing WSIS.

## 4. Experiments

In this section, we perform extensive experiments to evaluate the effectiveness of the proposed WS-RCNN, mainly including: (1) comparison with the state-of-the-art WSIS methods, both quantitatively and qualitatively; (2) validation of the effectiveness of some key components by comparing with variant baselines; (3) parameter study; (4) analysis on failure patterns. Our method was implemented in PyTorch on a workstation with 2 Nvidia Titan XP GPUs, Intel Core(TM) i7-8700 3.70 GHz CPU, 32 GB RAM and Ubuntu 18.04 OS.

### 4.1. Experimental Setup

#### 4.1.1. Datasets

Two well-known benchmarking datasets for instance segmentation, namely PASCAL VOC 2012 (termed as **VOC**) [53] and Microsoft COCO 2014 (termed as **COCO**) [54] are adopted for our experiments throughout this paper. **VOC** may be the most representative one for evaluating WSIS methods [8,13,33], which includes 10,582 images for training (*trainset*) and 1449 images for validation (*valset*) from 20 object classes. **COCO** is a much larger and more challenging dataset, including 82,783 images for training (*trainset*) and 40,775 images for validation (*valset*) from 80 object classes. This dataset has rarely been utilized for the task of WSIS before, and we consider it to enable more comprehensive evaluation. For both datasets, we take *trainset* for training and *valset* for testing, and no other annotations except for image-level class labels are used for training according to our problem definition of WSIS.

#### 4.1.2. Performance Metrics

We adopt $mAP_{r}$, the most commonly-used performance metric for instance segmentation [8,13,33,53], for quantitative evaluation and comparison, where *r* is the IoU threshold utilized to calculate the metric [53,55]. In our experiments on WSIS, we use $mAP_{50}$ (r=0.5) as the major metric for comparison and analysis, but we also report $mAP_{25}$ (r=0.25) and $mAP_{75}$ (r=0.75) for more in-depth evaluation.

#### 4.1.3. Implementation Details

We use VGG-16 [56] pre-trained on ImageNet as the backbone for the proposed WS-RCNN. The number of segment proposals per image is set to be N=200 following [8,13]. For training WS-RCNN, the SGD optimizer is used with a initial learning rate of $5\times 10^{-4}$ for the first $3.5\times 10^{4}$ iterations. In the following $10^{4}$ iterations, the learning rate decreases to $5\times 10^{-5}$. For data augmentation, we use five image scales {480,576,688,864,1200} (for the shorter side) with horizontal flips for both training and testing.

### 4.2. Comparison with State-of-the-Art

The study on WSIS is still at its early stage and there have not been many works so far. We consider four state-of-the-art WSIS methods in the literature for our comparative study, termed as **PRM** [8], **IAM** [13], **Label-PEnet** [9] and **IRnet** [14] respectively. For PRM [8] and IRnet [14], we use the source codes as well as configurations provided by the authors themselves. For IAM [13] and Label-PEnet [9], since no source codes are publicly released, we directly cite the results in the original literature [9,13] wherever available (marked with “-” if the results are unavailable). Besides, we also construct a variant of WSDDN [44], which is a very impactful method for weakly supervised object detection (rather than instance segmentation), termed as **WSDDN-seg**. For this sake, WSDDN-seg can be easily adapted to our task utilizes segments as the proposals to replace the original bounding-boxes in WSDDN (while keeping everything else unchanged). We consider WSDDN-seg because this method is spiritually similar to ours in that it also deploys a deep network to learn to score proposals. For fair comparison, we use the same method [51,52] to generate segment proposals for all the compared methods except IRnet [14], because the method focuses on improving the component of proposal generation. In addition, some methods perform post-processing (typically taking the obtained instance masks as pseudo annotations to run Mask R-CNN like [14]) to further refine the results while others do not. For fair and comprehensive comparison, we consider the two settings separately, i.e., without and with (marked by “+p”) using Mask R-CNN for refinement.

#### 4.2.1. Results on VOC

The quantitative results obtained by various methods on VOC are reported in Table 1. As can be observed, our WS-RCNN outperforms the state-of-the-art (IRnet [14]) by 11.6% in terms of the major metric $mAP_{50}$, which also achieves the best performance in terms of other metrics, either without or with refinement. Relative to the pioneering work of PRM [8], all the other methods make an improvement to an extent by enhancing certain aspects, including localization maps (IAM [13] and IRnet [14]), proposal generation (IAM [13]) and combination with object detection (Label-PEnet [9]). Comparatively, our WS-RCNN is able to improve by a far larger margin, demonstrating its effectiveness. Interestingly, one can observe that WSDDN-seg [44] can manage to achieve comparable or even better results than PRM [8]. Notice that WSDDN-seg exploits a CNN-based model to simply learn to score proposals (like ours), even without using any localization map at all. This may suggest that proposal scoring is indeed critical to WSIS with a large room for improvement, and “learning to score” is a promising strategy worthy of further exploration. We also show some comparisons of instance segmentation performance under different supervision on VOC in Table 2, including SDI [10], Mask R-CNN [2] and our WS-RCNN. SDI [10] uses bounding box supervision and Mask R-CNN [2] uses full supervision.

**Figure 5:**
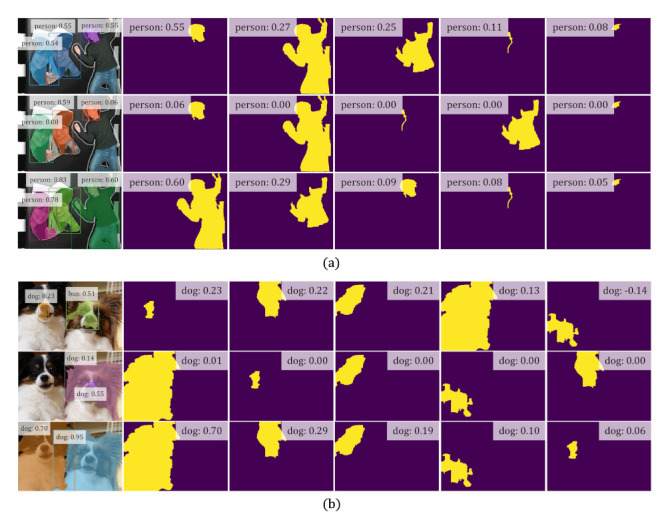
(**a**,**b**) are visualization of two sets of representative proposal scores obtained by various methods. In each set, from top to bottom are the results of PRM [8], WSDDN-seg [44] and our WS-RCNN respectively. In each row, the first column is the final WSIS result, and the second to sixth columns are the scores of the five proposals obtained by the method.

**Figure 6:**
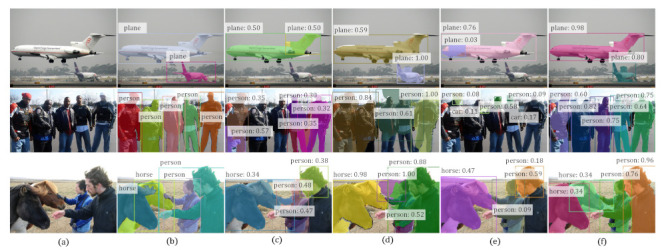
Representative results on VOC obtained by various methods. In each row, from left to right are (**a**) the input image, (**b**) the ground truth, and the results of (**c**) PRM [8], (**d**) IRnet [14], (**e**) WSDDN-seg [44] and (**f**) our WS-RCNN, respectively. Notice that we always output the same number of instances as that in the ground truth for all the methods to enable in-depth comparison.

To intuitively justify the motivation and merits of WS-RCNN, we comparatively visualize in Figure 5 two sets of representative intermediate results, namely, the scores of a same set of proposals acquired by PRM [8], WSDDN-seg [44] and our WS-RCNN (shown in the descending order of the proposal scores). One can observe that WSDDN-seg [44] tends to highlight one dominating instance while ignoring the others of the same class, and PRM [8] tends to highly score object parts or adjoining objects undesirably. Comparatively, our method can get more favorable proposal scores, which we argue is likely due to our advantageous learning mechanism. More representative results obtained by the various methods are further visualized in Figure 6.

#### 4.2.2. Results on COCO

**Figure 7:**
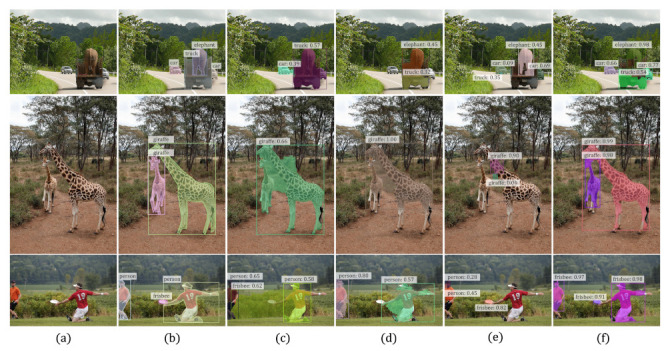
Representative results on COCO obtained by various methods. In each row, from left to right are (**a**) the input image, (**b**) the ground truth, and the results of (**c**) PRM [8], (**d**) IRnet [14], (**e**) WSDDN-seg [44] and (**f**) our WS-RCNN, respectively. Notice that we always output the same number of instances as that in the ground truth for all the methods to enable in-depth comparison.

Table 3 shows the quantitative results on the COCO dataset. Notice that COCO has not been considered in previous works compared, and the source codes of IAM [13] and Label-PEnet [9] are not publicly released, so the results for these two methods are unavailable. This dataset is far more difficult than VOC, as can be seen by the much worse overall performance of all the methods. However, our WS-RCNN can still outperform the compared methods, with a remarkable margin of 10.7% in terms of $mAP_{50}$ over the state-of-the-art (IRnet [14]), and 4.6% over WSDDN-seg constructed by ourselves. The relative performance of the compared methods in terms of other metric remains consistent with those on VOC, which further verifies the effectiveness of our approach. Some typical results are presented in Figure 7, which further demonstrate that our WS-RCNN can obtain more favorable instance masks, especially in case of multiple or adjoining instances.

### 4.3. Validation of Key Components

The superiority of the proposed WS-RCNN, to a large extent, should be attributed to the considerate design of its key components. We further carry out experiments on the VOC dataset to validate this point. For each component of concern, we construct some variants to replace the original one in WS-RCNN while keeping everything else unchanged, and compare the overall performance in terms of $mAP_{50}$.

#### 4.3.1. Pseudo Labeling Strategy

We consider the following three variants of AGPL:

- PLS-1: For each $p_{i}^{c}$ we assign those proposals which spatially include this point to the class *c*, i.e., assigning $z_{n}=c$ if $p_{i}^{c}\in R_{n}$, without considering the support mask $S_{i}^{c}$ (removing Line 5-8 in Algorithm 1 and meanwhile changing the condition in Equation (5) to be “$p_{i}^{c}\in R_{n}$” ).
- PLS-2: For each $p_{i}^{c}$ we thresold $M_{c}$ (using the threshold value of 0.5) and take the connected component surrounding this peak as the support mask $S_{i}^{c}$ (changing Line 6 in Algorithm 1).
- PLS-3: We simply adopt the method of PRM [8] for pseudo labeling, i.e., taking the proposal classification results obtained by the whole pipeline in [8] as the pseudo labels (changing Line 6 in Algorithm 1).

The results are reported in Table 4, which shows all these variants significantly underperform the proposed AGPL. The results validate the merit of AGPL, i.e., relying on peaks in attention maps and the spatial relationship between peak points and proposal regions to achieve pseudo labeling.

#### 4.3.2. Training Strategy

In our WS-RCNN, we adopt a particular two-phase strategy for network training (see Section 3.2. To verify the effectiveness of such a design, we construct two different training strategies for comparative study as follows:

- TS-coupled: When training the proposal scoring branch in the second phase, the backbone CNN is not reinitialized by the parameters pre-trained on ImageNet, but by those obtained during the training of the image-level classifier in the first phase.
- TS-joint: The image-level classifier is trained first, and the proposal scoring branch is trained jointly with the image-level classifier, i.e., combining the two training losses when training this branch.

The results in Table 5 demonstrate that the training strategy adopted by our WS-RCNN are more advantageous than the variants compared.

#### 4.3.3. Training Loss

To validate the effectiveness of our proposed EOSL loss, we compare it with a widely-used strategy for background handling as below

- LOSS-CE: We add a dummy class to accommodate background proposals and adopt the conventional binary cross-entropy to replace the EOSL loss in Equation (2).

As can be observed from Table 6 that our EOSL loss performs much better.

### 4.4. Parameter Study

Our approach involves specifying two parameters, i.e., the number of proposals *N* and the threshold value β in AGPL. Here we further conduct experiments to analyze the impact of these parameters on performance.

#### 4.4.1. The Number of Proposals *N*

**Figure 8:**
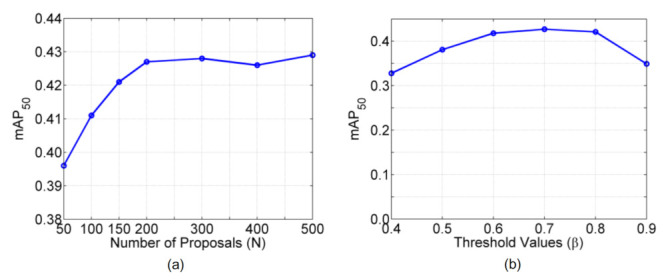
Impacts of varying the key parameters (**a**) the number of proposals *N* and (**b**) the threshold value β in AGPL.

We vary the number of proposals to be N={50,100,150,200,300,400,500}, and report the results in Figure 8a. As can be seen, the performance will not increase significantly after N>200, and we adopt N=200 throughout our experiments for the best tradeoff between performance and efficiency.

#### 4.4.2. The Threshold β in AGPL

We vary the threshold value to be β={0.4,0.5,0.6,0.7,0.8,0.9}, and the results are depicted in Figure 8b. One can observe that the performance is relatively stable when β∈[0.5,0.8] and we choose a fixed value of β=0.7 throughout our experiments.

### 4.5. Failure Cases

**Figure 9:**
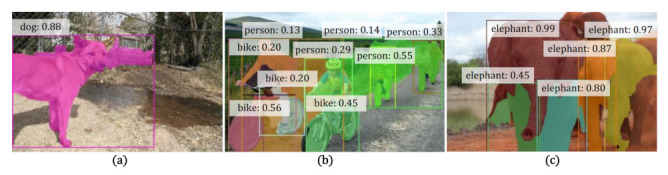
Typical failure cases of WS-RCNN. (**a**) Poor proposals. (**b**) Picking up large proposals covering multiple instances. (**c**) Picking up small proposals covering only a part of a instance.

Despite the effectiveness of WS-RCNN, WSIS is essentially a challenging task and the overall performance still has much room for improvement. Here we show several typical failure cases of WS-RCNN in Figure 9. Since our method is proposal-based, it relies much on the quality of proposals. If the proposal generator fails to cover the spatial extents of the true instance, our method will fail consequently (see Figure 9a). Another typical failure case is that if there are a number of instances of a class with overlap among or close to each other, our method may pick up those large proposals which cover multiple instances (see Figure 9b) or the small proposals which cover only a part of a instance (Figure 9c) (Notice that the desired proposals are present in (b) and (c).

## 5. Conclusions

In this paper, we have presented a simple, natural but surprisingly effective framework, termed as Weakly Supervised R-CNN (WS-RCNN), for weakly supervised instance segmentation (WSIS). The basic idea is to deploy a deep network to learn to score proposals under the particular setting of weak supervision. For this sake, a strategy called Attention-Guided Pseudo Labeling is proposed to address the key issue of proposal-level pseudo labeling. And a so-called Entropic Open-Set Loss is introduced for model training to further improve the robustness. Comprehensive experiments on two well-known datasets, i.e., PASCAL VOC 2012 and Microsoft COCO 2014, have demonstrated that the proposed WS-RCNN can significantly outperform the state-of-the-art. Experiments have also been carried out to validate effectiveness of the key components and to study the impacts of some key parameters. In our future work, we will consider integrating a learning-based proposal network to replace the current heuristic proposal generator in WS-RCNN in order to alleviate its dependency on the quality of proposals. We will also explore how to further improve our approach in dealing with complex scenes (e.g., complex background, multiple or adjoining instances of the same class) since there is generally much room for improvement.

## Figures and Tables

**Table 1:** Quantitative results obtained by various WSIS methods on VOC.

Methods	mAP_50_	${\mathrm{mAP}}_{25}$	${\mathrm{mAP}}_{75}$	${\mathrm{mAP}}_{50}\left(+p\right)$	${\mathrm{mAP}}_{25}\left(+p\right)$	${\mathrm{mAP}}_{75}\left(+p\right)$
PRM [8]	26.8	44.3	9.0	38.0	52.8	14.1
IAM [13]	28.8	45.9	11.9	-	-	-
Label-PEnet [9]	30.2	49.1	12.9	-	-	-
IRnet [14]	31.1	49.2	10.7	46.7	60.5	15.6
WSDDN-seg [44]	27.5	47.5	9.8	43.7	55.9	16.9
WS-RCNN (ours)	42.7	57.2	19.4	47.3	62.2	19.8

**Table 2:** Instance segmentation performance under different supervision on VOC.

Method	Supervision	${\mathrm{mAP}}_{50}$	${\mathrm{mAP}}_{25}$	${\mathrm{mAP}}_{75}$
SDI [10]	Box-Level	44.8	-	16.3
Mask R-CNN [2]	Fully-Supervised	69.0	76.7	52.5
WS-RCNN	Image-Level	47.3	62.2	19.8

**Table 3:** Quantitative results obtained by various WSIS methods on COCO.

Methods	mAP_50_	${\mathrm{mAP}}_{25}$	${\mathrm{mAP}}_{75}$	${\mathrm{mAP}}_{50}\left(+p\right)$	${\mathrm{mAP}}_{25}\left(+p\right)$	${\mathrm{mAP}}_{75}\left(+p\right)$
PRM [8]	5.8	12.1	1.8	14.7	23.7	6.6
IRnet [14]	7.3	13.9	2.4	13.2	22.2	4.8
WSDDN-seg [44]	14.4	24.0	6.0	23.0	30.9	11.5
WS-RCNN (ours)	18.0	27.4	7.2	24.2	32.1	11.6

**Table 4:** Comparison with Various Pseudo Labeling Strategies on VOC.

PLS-1	PLS-2	PLS-3	Ours
24.0	25.2	36.2	**42.7**

**Table 5:** Comparison with Various Network Structures and Training Strategies on VOC.

TS-Coupled	TS-Joint	Ours
36.5	37.6	**42.7**

**Table 6:** Comparison with Different Training Loss for Background Handling on VOC.

LOSS-CE	Ours
34.5	**42.7**

## Data Availability

The study did not report any data.
